# Characterisation of the complete chloroplast genome of the common bean, *Phaseolus vulgaris* L.

**DOI:** 10.1080/23802359.2018.1502634

**Published:** 2018-08-20

**Authors:** Xianxin Meng, Ming Li

**Affiliations:** a College of Agriculture, Northeast Agricultural University, Harbin, People’s Republic of China;; b Crop Breeding Institute, Heilongjiang Academy of Agricultural Sciences, Harbin, People’s Republic of China

**Keywords:** *Phaseolus vulgaris* L., common bean, chloroplast genome, phylogenetic analysis, HomBlocks

## Abstract

Common bean (*Phaseolus vulgaris* L.) is the most important grain legume in the human diet and has a role in sustainable agriculture. In this study, we obtained the complete chloroplast genome sequence of common bean using BGISEQ-500 sequencing. Its chloroplast genome size has 155,622 bp, containing a large single copy region (84,924 bp), a small single copy region (18,570 bp) and a pair of IR regions (26,064 bp). The overall GC contents of the chloroplast genome were 37.9%. This circular genome contains 131 annotated genes, including 85 protein-coding genes, 37 tRNAs, and 8 rRNAs. The phylogenetic analysis using maximum likelihood (ML) method showed that Common bean has the closest relationship with *Phaseolus coccineus L.* This complete chloroplast genomes can be subsequently used for the genetic breeding and agricultural development of this valuable species.

Common bean (*Phaseolus vulgaris* L.) is the important crop in the family legume. It is a major source of dietary protein throughout both Latin America and Eastern Africa. It has been cultivated in Latin America for more than 7000 years and China has abundant resources as secondary origin center (Graham and Ranalli 1997). The species belongs to a large and diverse genus that comprises 70–80 species, five of which have been domesticated (Bitocchi et al. 2013). Among these domesticated species, common bean is the one with the broadest geographic distribution and the highest agronomic, nutritional and economic value (Ariani et al. 2016). However, our understanding of the origins of the chloroplast genome of this crop plant is limited. In this study, we obtained the complete chloroplast genome of *P. vulgaris* L. and explored the phylogenetic relationship with other species, which contributes to phylogenetic studies of these taxa and the sustainable agricultural development of this valuable species.

The specimen of *P. vulgaris* L. was isolated from Heilongjiang Academy of Agricultural Sciences Minzhu test field in Harbin, Heilongjiang, China (126.36E; 45.40N). The total DNA of *P. vulgaris* L. was extracted using CTAB method and stored in Heilongjiang Academy of Agricultural Sciences Crop Breeding Institute (No. HAASCBI01). The DNA sample was sequenced using the BGISEQ-500 Sequencing Platform (BIG, Shenzhen, China). Quality control was performed to remove low-quality reads and adapters using the FastQC software (Andrews 2015). The chloroplast genome was assembled with SPAdes version 3.8 (St Petersburg, Russia) (Bankevich et al. 2012) and annotated by Blast and DOGMA (Wyman et al. 2004). The tRNA genes were further identified using tRNAscan-SE 2.0 (Lowe and Chan 2016). The annotated chloroplast genome was submitted to GenBank database under accession No. MH553374.

The complete chloroplast genome of *P. vulgaris* L. was a circle with 155,622 bp in size, containing a large single copy region (LSC) of 84,924 bp, a small single copy region (SSC) of 18,570 bp and a pair of inverted repeat regions (IRs) of 26,064 bp. In total 131 genes were annotated on this chloroplast genome, including 85 protein-coding genes (PCG), 37 transfer RNA genes (tRNA) and 8 ribosomal RNA genes (rRNA). In the IR regions, a total of 17 genes were found duplicated, including 7 PCG species (*rpl2, rpl23, ycf2, ycf15, ycf1, rps7* and *ndhB*), 6 tRNA species (*trnI-GAU, trnL-CAA, trnV-GAC, trnI-CAU, trnA-UGC, trnR-ACG* and *trnN-GUU*) and 4 rRNA species (*rrn4.5, rrn5, rrn16* and *rrn23*). The overall nucleotide composition is: 30.8% A, 31.4% T, 19.3% C, and 18.6% G, with a total G + C content of 37.9%.

For phylogenetic analysis, we selected other 44 plants chloroplast genomes from GenBank to assess the relationship of *P. vulgaris* L. The genome-wide alignment of all chloroplast genomes was constructed by HomBlocks (Bi et al. 2017). The phylogenetic trees were reconstructed using maximum likelihood (ML) methods. ML analysis was performed using RaxML-8.2.4 (Stamatakis 2014), of which the bootstrap values were calculated using 5000 replicates to assess node support and all the nodes were inferred with strong support by the ML methods. The final tree was represented and edited using MEGA (Kumar et al. 2018). As shown in the phylogenetic tree (Figure 1), the chloroplast genome of *P. vulgaris* L. was clustered with *Phaseolus coccineus L.*

**Figure 1.**
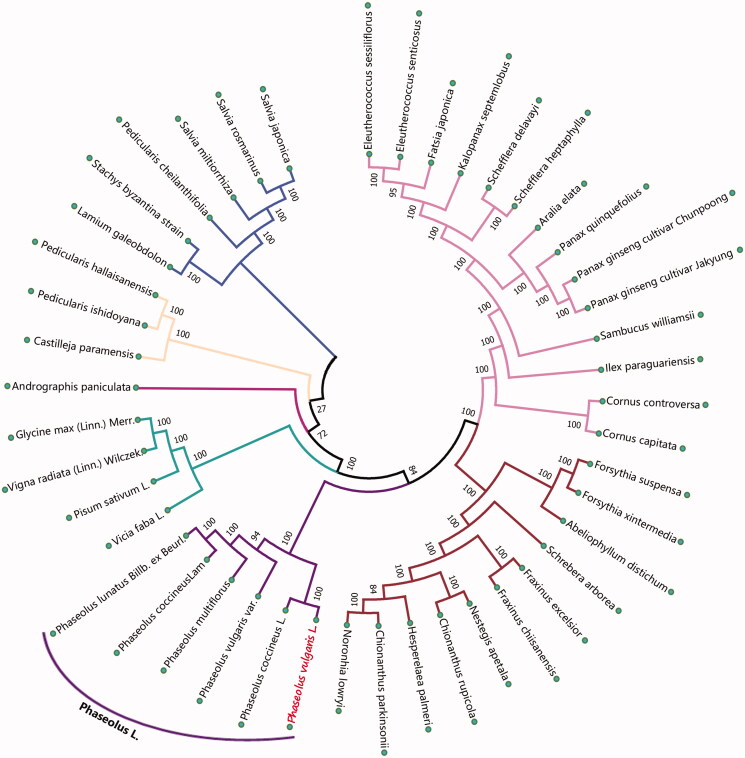
The maximum likelihood (ML) tree inferred from 45 plants chloroplast genomes. This tree was drawn without setting out groups. All nodes exhibit above 90 % bootstraps. The length of branch represents the divergence distance.
